# Supplementary material for: Contamination by Trace Elements and Oxidative Stress in the Skeletal Muscle of Scyliorhinus canicula from the Central Tyrrhenian Sea
Source: Antioxidants (Basel). 2023 Feb 19;12(2):524. doi: 10.3390/antiox12020524 (PMC9952106; doi:10.3390/antiox12020524)
Supplement: Supplementary file 1 [file antioxidants-12-00524-s001.zip › antioxidants-2219228-supplementary.pdf]

Table S1. Biological and biometric data of the 24 specimens of *S. canicula* enrolled for the present study. All individuals were sexually mature.

Sample code	Sex	Weight (g)	Length (cm)
SCI17004	F	154	33
SCI18040	F	200	40
SCP18054	F	185	42
SCI18039	F	180	38
SCI17007	M	317.5	42
SCE17024	M	160	36
SCE17025	M	175	41
SCI18029	F	245	44
SCI17005	F	263	39
SCI17010	F	199.6	35
SCI18034	M	270	43
SCP18056	F	175	39.5
SCP18053	F	170	40
SCE17023	F	135	37
SCP18052	M	255	47
SCP18055	F	160	39
SCP18058	M	140	39
SCI17019	F	317.5	40
SCP18057	F	165	40
SCI18030	F	195	40
SCP18060	F	180	41.5
SCP18051	F	205	42
SCP18059	F	155	39
SCI18036	F	200	37.5
